# Effects of dietary management for medium-chain acyl-CoA dehydrogenase deficiency (MCADD) on eating behaviour in childhood, adolescence and young adulthood

**DOI:** 10.1186/s40795-025-01209-9

**Published:** 2025-11-14

**Authors:** Pauline Banaszak, Nadja Wolke, Anja Markant, Ulrike Och, Frank Rutsch, Tobias Fischer

**Affiliations:** 1 Center for Nutrition and Therapy (NuT), University of Applied Sciences Muenster, Corrensstraße 25, Muenster, 48149 Germany; 2 Department of Paediatrics, University Hospital Muenster, Albert-Schweitzer-Campus 1, Muenster, 48149 Germany

**Keywords:** Eating behaviour, Eating disorders, Eating problems, Dietetics, Dietetic restrictions, Inherited errors of metabolism, Inborn errors of metabolism, Medium chain acyl-CoA dehydrogenase (MCAD), Medium chain acyl-CoA dehydrogenase deficiency (MCADD)

## Abstract

**Background:**

Medium-chain acyl-CoA dehydrogenase deficiency (MCADD) is an autosomal recessive disorder of ß-oxidation. A loss of enzyme function results in impaired breakdown of medium-chain fatty acids. The main treatment involves a dietetic approach to ensure adequate food intake and avoiding fasting. Diseases treated with a strict diet during childhood are often associated with eating problems. However, research in this area is very limited. This exploratory observational study aimed to examine the effects of dietary management and the underlying recommendations for MCADD on eating behaviour, as well as their subsequent impact on family life.

**Methods:**

Paediatric and young adult patients (age 8–25) with MCADD and their caregivers at the University Hospital Muenster were included. A descriptive semi-quantitative study design incorporating qualitative elements was chosen. A novel combination of questions from validated questionnaires on eating behaviour was used to collect quantitative and qualitative data. Data were collected via a combination of telephone interviews and online surveys.

**Results:**

Of the 28 patients contacted, 13 (46%) were successfully recruited into the study. The results showed that most of the study participants did not exhibit abnormal eating behaviour. Additionally, the patients did not show an increased prevalence of being overweight at the time of data collection. A higher temporary tendency to be overweight was only observed when considering the overall weight trend. Dietary interventions placed a higher burden on family life in infancy (0–4 years) than at the time of the survey. Maintaining a safe fasting interval was the main reason for restrictions in family life.

**Conclusion:**

This observational and exploratory sample showed no tendency towards abnormal eating behaviour associated with dietary management in MCADD.

**Supplementary Information:**

The online version contains supplementary material available at 10.1186/s40795-025-01209-9.

## Introduction

Congenital deficiency of the enzyme medium-chain acyl-CoA dehydrogenase (MCAD) (ICD-10: E71.3; OMIM: 201450) is one of the most common disorders of ß-oxidation in Germany with an prevalence of 1:10,607 (*n* = 75) in 2021 and 1:11,273 (*n* = 65) in 2022, according to the national screening reports [1–3]. and is mainly diagnosed by German extended newborn screening [4]. MCAD deficiency (MCADD) is an autosomal recessive inherited defect of the medium-chain acyl-CoA dehydrogenase gene (ACADM; 60700) on chromosome one (1p31) [5]. Loss of MCAD function leads to impaired degradation of the medium-chain fatty acids such as caproic acid (C6:0), caprylic acid (C8:0), capric acid (C10:0) and lauric acid (C12:0). This means that fatty acids cannot be utilised sufficiently for energy generation, since long-chain fatty acids are only broken down to the specified chain length of MCAD in the oxidation cycle [6]. The first clinical symptoms typically appear within the first 24 months postnatally and are associated with the occurrence of prolonged periods of fasting. Causes of prolonged fasting include, for example, weaning babies from night-time feeding or acute infections that are accompanied by fever, loss of appetite and an increased need for energy [7]. Clinical features of acute energy deficiency include hypoketotic hypoglycaemia in particular, which can be accompanied by many other symptoms such as vomiting, lethargy, seizures, encephalopathies and, in rare cases, arrhythmias [7–10]. If left untreated, an MCADD crisis can quickly lead to complete metabolic decompensation, coma and death [11]. Early intervention by specialised metabolic centres can reduce morbidity and mortality to a very low level [12].

As MCADD is a dietary condition, early diagnosis and consistent adherence to dietary guidelines are crucial for the good development of those affected [13]. To meet metabolic requirements, sufficient food intake has to be ensured and fasting must be avoided [14]. Due to existing fasting intolerance, infants must be fed at least every four hours. Between 6 and 12 months of age, there should be no more than an eight-hour gap between meals. In the second year of life, this should be no more than 10 h and, after the age of two, no more than 12 h [15]. However, these times only apply in a metabolically balanced state. If these feeding intervals are not maintained, or if there is a risk of metabolic imbalance, hospital treatment involving a glucose-containing infusion should be administered immediately. The diet for patients with MCADD typically aligns with national reference values for nutrient and energy intake, and no special criteria are required. Nevertheless, a slightly low-fat diet that falls below the fat reference intake in national dietary guidelines is often recommended [7]. Intake of medium-chain triglycerides (MCTs) is contraindicated, and products or infusions enriched with MCTs must be avoided [16].

Studies on the management of chronic diseases through diet, such as coeliac disease, type 1 diabetes mellitus, cystic fibrosis, inflammatory bowel disease and other inherited metabolic disorders, indicate that an intense focus on nutrition, strict parental control and limited personal control over dietary choice can increase the risk of developing disordered eating behaviours [8–10, 17–20]. Furthermore, patients with MCADD often tend to be overweight, which is also a risk factor for the development of disordered eating behaviour [7, 21–23]. Disordered eating behaviours can significantly impact on both physical and mental health. This highlights the importance of identifying potential risk groups early on to enable timely intervention [24, 25]. In this context, this study investigated the effects of dietary interventions and underlying recommendations for MCADD on eating behaviour focusing on the potential link between dietary interventions and the development of potential eating problems. Furthermore, the study aimed to demonstrate possible consequences for weight development and analyse the burden and restrictions on family life due to dietary considerations.

## Methods

### Online questionnaire and telephone interview

A descriptive semi-quantitative study design with qualitative options was chosen. MCADD patients at the University Hospital Muenster (UKM) and their parents were included. In addition to having MCADD, participants were required to have an adequate knowledge of the German language and be willing to participate. There were no further inclusion or exclusion criteria. The selection of participants was based on a total sampling of all patients with MCADD followed at the UKM on a voluntary basis. Paediatric Dietetics Department at UKM and the study team invited patients to participate by sending them postal or telephone information about the study. Both the test participants and their legal guardians provided written consent after receiving comprehensive information about the study details. Children and adolescents were given age-appropriate information about the research project in simple language, both verbally and in writing.

Data were collected using a combination of telephone interview and an online survey. In total, there were 55 questions, 28 of which were asked via the online questionnaire and 27 via the telephone interview (see Supplement S1.1 and S1.2). The questions were based on the Eating Attitude Test (EAT-26), the Intuitive Eating Scale-2 (IES-2), the Adult Eating Behaviour Questionnaire (AEBQ), and Schweiker’s (2016) questionnaire [26–29]. The selection of items was hypothesis-driven, aiming to capture potential eating-related issues in individuals with MCAD across different domains, such as body perception, intuitive eating, general eating behaviour. Where available, validated German versions were used (e.g., EAT-26, IES-2 [30, 31]); AEBQ items were translated into German. The combined tool is used to identify tendencies towards eating problems, but it is not intended for diagnosing eating disorders.

More sensitive questions, such as `I keep eating, even though I am already full, because I think I have to eat it all.´ (Question 23) were integrated into the online questionnaire in order to minimise the influence of social desirability [32]. In addition, the online questionnaire included metric and closed questions, such as information on date of birth, height and weight. The online survey was conducted using the survey tool LimeSurvey (LimeSurvey GmbH, Hamburg, Germany). The recorded telephone interview included open-ended questions that allowed participants to describe their personal experiences in more detail. The responses to these questions were grouped post hoc using a simple, descriptive categorisation. In addition to the child, at least one parent was interviewed, and care was taken to ensure that the parents’ answers did not influence the child by delaying the interview. The questions were organised into thematic areas related to eating behaviour and the disorder itself.

Prior to the two pre-tests, a multidisciplinary team (including a nutritionist, dietitian and psychologist) reviewed the compiled questionnaire. Subsequently, two pre-tests were conducted with non-specialists and specialists (nutritionists and a medical doctor) to ensure that the questionnaire was comprehensible and content-valid. The questionnaire was revised based on feedback from both groups. No further reliability measurements or validity tests were carried out. The study protocol was approved by the Ethics Committee of the Medical Association of Westphalia-Lippe and the University of Muenster (project identification code: 2023-392>-f-S) in accordance with the Declaration of Helsinki.

### Data analysis

Responses from the online survey were imported into Microsoft Excel 365 and IBM SPSS 29 from LimeSurvey, while the responses from the structured telephone interviews were transferred manually. All quantitative data were first analysed using descriptive statistics such as minimum, maximum, mean, median and standard deviation (SD). IBM SPSS Statistics 29 was used for advanced data processing. Body mass index (BMI; kg/m²) was calculated from the anthropometric data of height (m) and weight (kg). The anthropometric data were compared with the reference percentiles according to Kromeyer-Hauschild et al. [33]. To represent age-specific subgroups, the sample was divided into two groups: children aged 8 to 13 years, and adolescents and young adults aged 14 to 20 years. Fisher’s exact test was used to determine significant differences in responses to questions 15–28, 34 and 37 between the age groups and gender. Due to the small sample size, some of the responses were combined into ‘(rather) true’ and ‘(rather) not true’ categories for the statistical analysis. The Mann-Whitney-U test was used to analyse the differences between the ratings given by children and parents in relation to eating behaviour, food restrictions, and the burden on family life. Wilcoxon signed-rank test was used to compare the responses of parents regarding questions about their child’s eating behaviour restrictions (45, 45b) and related family life restrictions (46, 46b) between the current situation and in early childhood (0–4 years of age). The significance level was *p* < 0.05. Due to the limited number of cases, it was not possible to form additional subgroups based on factors such as educational background or BMI.

## Results

13 of the 28 patients contacted (46%) were successfully recruited into the study. Participants ranged in age from 8 to 20 years (14.3 ± 3.9 years). 5 participants (38.5%) were in the 8–13 age group, while 8 (61.5%) were in the 14–20 age group. This age range enabled a retrospective longitudinal analysis, which illustrated the evolving experiences of both the participants and their parents over time. 46.2% of participants were female and 53.8% were male (see Table 1). The online questionnaire took 5 to 10 min to complete (7.9 ± 2.7 min), depending on the age of the participants, while the telephone interview took an average of about 30 min (30.4 ± 6.8 min).

**Table 1:** Demographic and anthropometric characteristics of the study participants with MCADD at the time of data collection (*n* = 13)

Age		14,31 ± 3,9
Sex	Female	46,2% (*n* = 6)
	Male	53,8% (*n* = 7)
BMI Percentile	≥ P97	7,7% (*n* = 1)
	P90 - < P97	7,7% (*n* = 1)
	P75 - < P90	30,8% (*n* = 4)
	P50 - < P75	30,8% (*n* = 4)
	P25 - < P50	7,7% (*n* = 1)
	< P25	15,4% (*n* = 2)

### Exercise behaviour and weight development

**Fig. 1:**
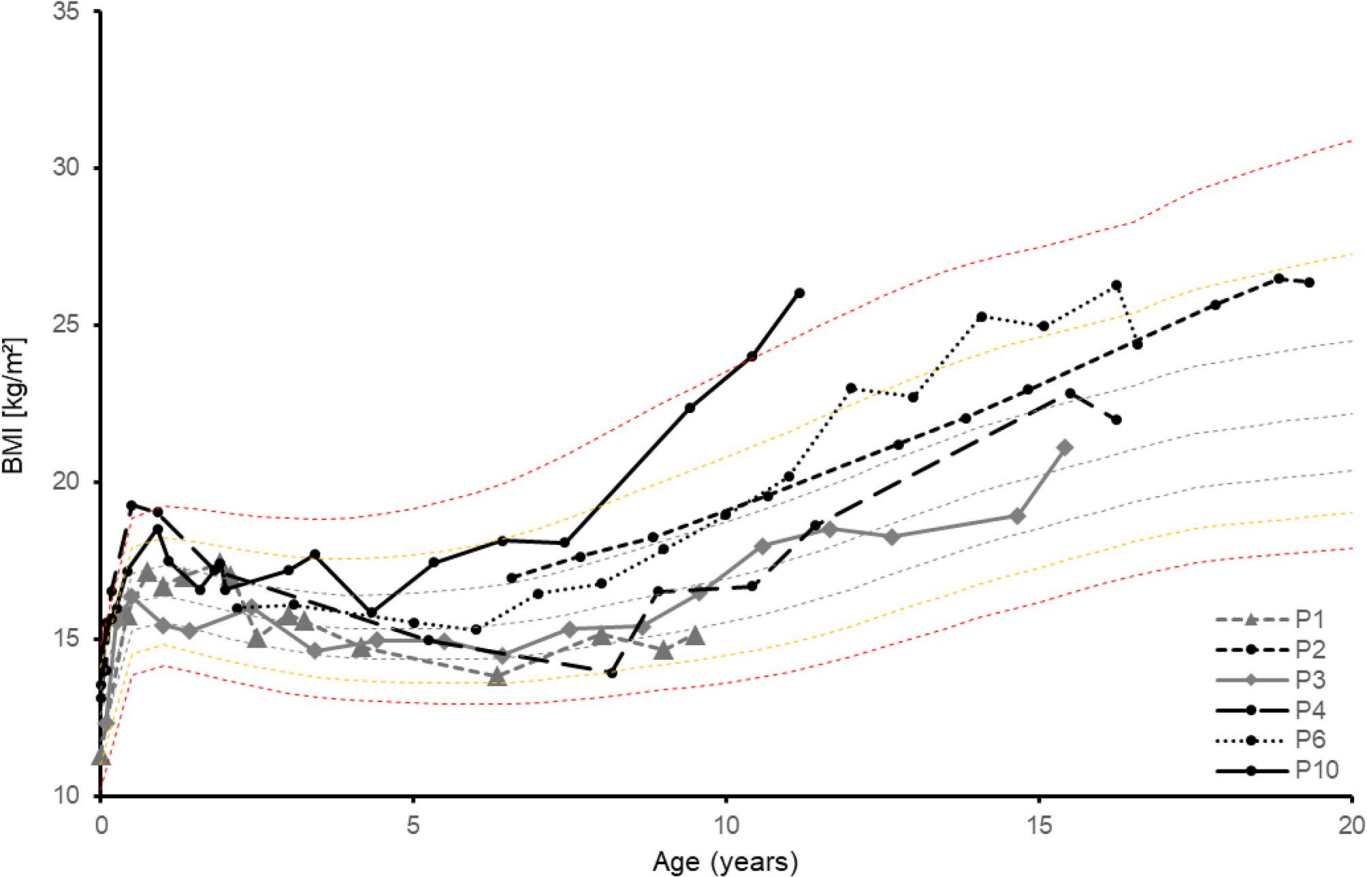
BMI-progression depending on the age of the female participants (0–19 years, *n* = 6) compared to the percentile curves according to Kromeyer-Hauschild et al. (2001); *P* = patient [33]

**Fig. 2:**
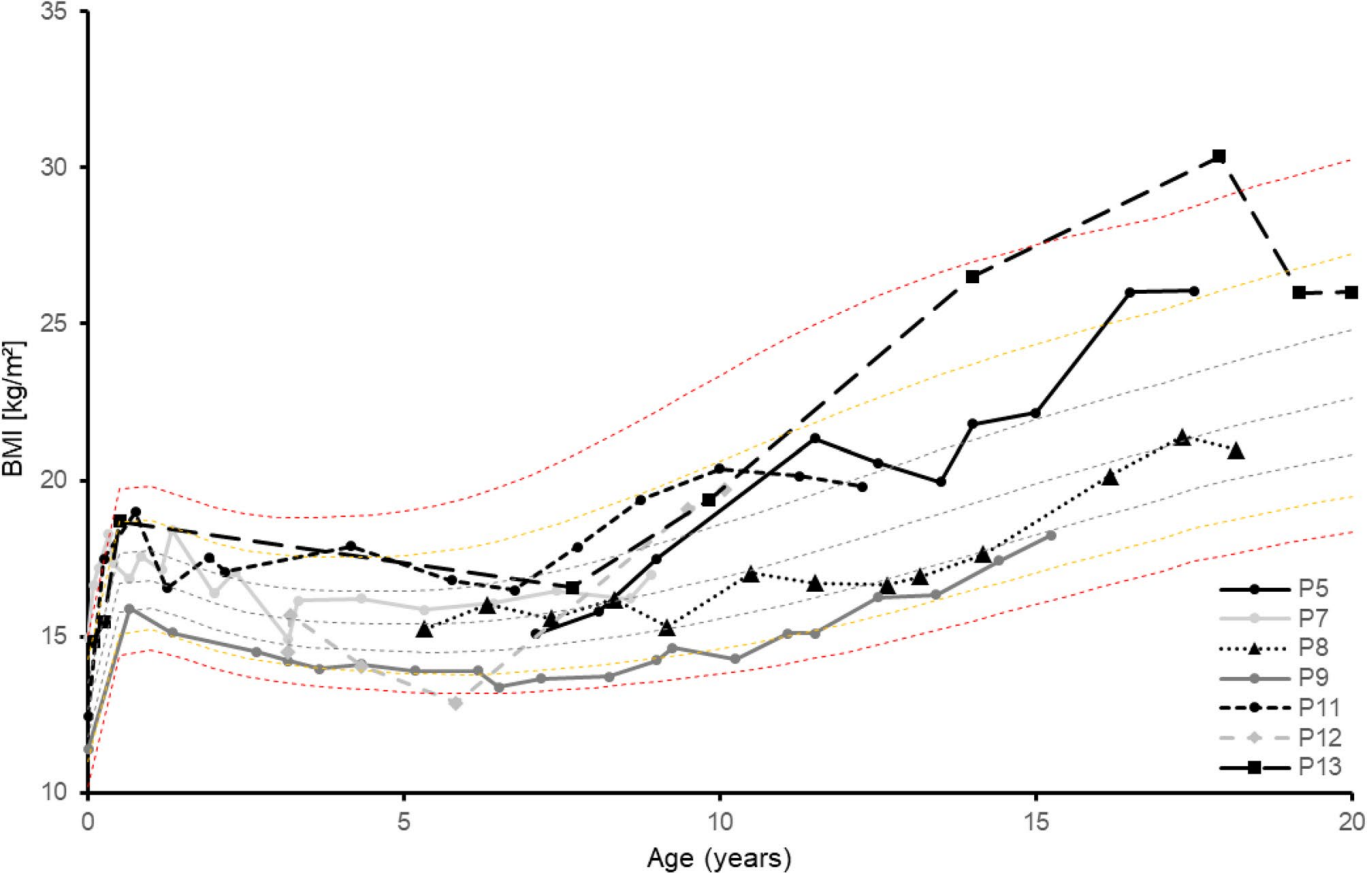
BMI-progression depending on the age of the male participants (0–20 years, *n* = 7) compared to the percentile curves according to Kromeyer-Hauschild et al. (2001); *P* = patient [33]

The majority of subjects (92.3%) exercised regularly. At the time of completing the questionnaire, the BMI was below the 25th percentile in 15.4% (*n* = 2), below the 50th percentile in 7.7% (*n* = 1) and above the 50th or 75th percentile in each 30.8% (*n* = 4) of participants. In 7.7% (*n* = 1) of the cases the BMI was above the 90th (P90) or 97th (P97) percentile. The prevalence of obesity tended to increase with age. Contrary to the time of the questionnaire, the available anthropometric data from the entire care period showed that a total of four subjects had a BMI above the 90th and 97th percentiles at certain time points (see Figs. 1 and 2). Taking the overall weight progression into account, there was higher temporary tendency (30.8%) to be overweight or obese (see Figure S3.1 and S3.2).

### Eating behaviour

Most participants (92.3%, *n* = 12) regularly ate three meals a day. The frequency of snacks varied from 1 to 2 (30.8%, *n* = 4), 1 to 3 (23.1%, *n* = 3) and 2 to 3 (30.8%, *n* = 4) a day. Both the participants (question nr. 33; S1.2) and their parents (question nr. 44; S1.2) were asked whether any restrictions, special features or dietary requirements were currently being observed in their eating behaviour. 50.0% of the participants and 46.2% of the parents confirmed this, however only 38.5% of the children’s and parents’ responses matched (*p* = 0.851). Differences were particularly noticeable among older participants (17–20 years). 33.3% (*n* = 2) of participants reported eating more fruit and vegetables and 33.3% (*n* = 2) reported eating fewer sweets. One further participant reported eating more fruit. One participant each reported eating less coconut fat, sufficient calories, protein and carbohydrates. Among parents, 33.3% (*n* = 2) paid attention to low coconut fat/palm fat and 16.7% (*n* = 1) to calorie requirements, less sweets and more fruit and vegetables.

All parents stated that they observed dietary guidelines during the infant stage (0–4 years), and there was a significant difference (*p* = 0.008) between the current situation and the earlier years of the participants with regard to parental adherence to certain dietary guidelines. All parents paid attention to a strict regularity of food intake (100%, *n* = 13). 30.8% (*n* = 4) made sure the diet was rich in carbohydrates. 23.1% (*n* = 3) ensured their children consumed sufficient food in the evening, while 15.4% (*n* = 2) avoided fried foods, minimized coconut fat and excluded chocolate from their children’s diet.

### Influence of dietary management on eating behaviour

**Fig. 3:**
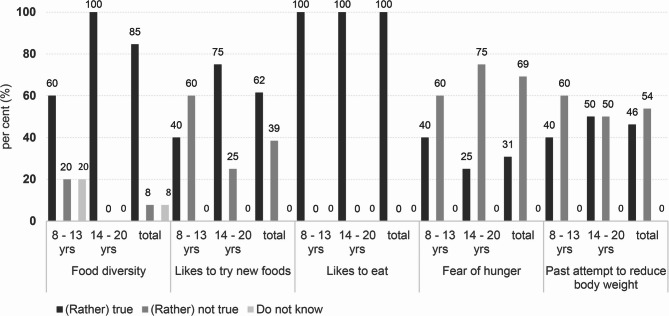
Responses to selected questions (in percentage) about potential problems with eating behaviour of all MCADD patients included in the study (total, *n* = 13) and categorised by age groups (8–13 [*n* = 5] and 14–20 [*n* = 8] life years [yrs])

84.6% (*n* = 11) of respondents reported enjoying a wide range of foods, 61.5% (*n* = 8) expressed a willingness to try new things, and 92.3% (*n* = 12) allowed themselves to eat whatever they desired. All respondents enjoyed eating. However, insecurities were also evident: 23.1% (*n* = 3) ate even when they were not hungry because they were worried about health consequences. 38.5% (*n* = 5) expressed fear of gaining weight and just under half (46.2%, *n* = 6) had already tried to lose weight by eating less (see Fig. 3). Differences between age groups and genders were apparent but not statistically significant, particularly in relation to body confidence and dieting behaviour (see Supplement S2).

### Influence of the disease on eating habits and family life

92.2% (n = 12) of parents reported that their parenting had been influenced their child’s illness, particularly with regard to nutrition. Parents described that the children were made aware of the importance of eating regularly and of eating when unwell. 33.3% (n = 4) stated that sleeping in was not permitted or that food was brought to the children in bed in the morning. Similarly, 33.3% (n = 4) saw an impact on kindergarten and school time, as the children were made aware of how to act in the event of health problems. 41.7% (n = 5) emphasised the importance of educating children about snacks, rest, the risk of injury during sports, and how to deal with alcohol in adolescence.

76.9% (n = 10) of parents reported that the illness had a negative impact on family life, particularly with regard to holidays and vacation planning. Families tended to holiday in Germany and chose their vacation destination based on the availability of medical care. 50% (n = 5) reported increased worries, particularly concerning their children’s diet and sports activities. 40% (n = 4) found that periods of illness led to increased anxiety and tension, while the same proportion described how everyday family life was dominated by the responsibility of caring for a child with a metabolic disorder.

At the time of the survey, the majority of parents (76.9%, *n* = 10) did not feel restricted in their daily eating behaviour. 7.7% (*n* = 1) felt moderately restricted and 15.4% (*n* = 2) felt slightly restricted, mainly because of the effort to ensure regular and balanced meals and conflicts when the child did not want to eat. In the first four years of life, 30.8% (*n* = 4) of the parents felt severely restricted, another 30.8% (*n* = 4) felt moderately restricted and 23.1% (*n* = 3) felt slightly restricted. The main reasons were keeping to safe fasting times (72.7%, *n* = 8), waking children at night and the associated sleep deprivation and refusal to eat (36.4%, *n* = 4). Other reasons included single parenthood, anxiety and psychological stress. A comparison of the current feelings and the ones experienced in infancy showed significant differences for both cases in terms of limitations in daily life (*p* = 0.004) and strain on family life (*p* = 0.004).

## Discussion

The aim of this study was to investigate the potential link between dietary management and abnormal eating behaviour in children and adolescents with MCADD. A descriptive, semi-quantitative approach combining telephone interviews and an online questionnaire was adopted.

Maintaining a consistent safe fasting interval and sufficient energy intake in MCADD can increase the risk of developing obesity in those affected. The German Health Interview and Examination Survey for Children and Adolescents (KiGGS) wave 2 (2014–2017; *N* = 13,568) showed that, according to the reference system of Kromeyer-Hauschild et al. (2015), 15.4% of children and adolescents (aged 3–17) in Germany are categorised as overweight or obese [34, 35]. The present study corresponds to this range and initially showed no abnormalities. However, the patients’ weight histories revealed a higher tendency to be overweight. This is also confirmed by the proportion of subjects with BMI values that exceed the 50th percentile (77% insted of 50%) and he 75th percentile (46.2% insted of 25%) base on the BMI percentiles according to Kromeyer-Hauschild et al. (2015) [35]. Accordingly, the additional consideration of BMI trajectories shows a higher temporary prevalence of overweight over time (see Figs. 2 and 3) and confirms the findings of Derks et al. (2006) that young people with MCADD have a higher risk of being overweight [36].

### Impact on eating behaviour

The study of eating behaviour showed that the number of main meals and snacks per day consumed by the surveyed MCADD patients did not differ from the usual recommendations for meal frequency [37, 38]. The interview results also indicated that most meals were enjoyed together with the family at the table. A family eating structure involving regular meals together has been associated with healthy eating patterns and a low BMI, which could reasonably explain this finding [39]. This study identified age-related differences in possible specificities of eating behaviour. Participants in the 17–20 age group reported paying attention to certain aspects of their eating behaviour, such as controlling energy intake. Parents of participants in this age group did not provide this information, suggesting greater degree of independence in this age group. The opposite was true in the 8–15 age group. While children in this age group denied paying attention to certain aspects of their own eating behaviour, parental control was greater. Piercy et al. (2021) (10–15 yrs, *n* = 12) corroborate these findings by reporting greater independence and a stronger sense of responsibility among participants in advanced puberty at 14–15 years (*n* = 3) compared to the younger participants (10–13 years, *n* = 9). However, Piercy et al. (2021) also reported strong parental involvement in food choices and encouragement to eat regularly among the 14–15 age group [40]. These findings suggest that, as with adolescents with other metabolic disorders, a developmental process towards self-regulation of dietary behaviour also progresses with age in people with MCADD [41, 42].

Of the participants who attempted to maintain a moderate energy intake (15,4%, *n* = 2), all had a BMI at or above the 75th percentile. This suggests that both the participants and their parents were aware of the obesity risk. If parental behaviour signals that the child should eat less or is perceived as overweight, this can lead to body dissatisfaction and to the development of problematic dieting behaviour in children and adolescents [43–46]. Both are considered predictors of future weight gain and should therefore be addressed in clinical practice [47, 48].

The questions about existing eating problems showed no tendency towards abnormal eating behaviour. Most participants ate a wide variety of foods (84.6%, *n* = 11), enjoyed trying new foods (61.5%, *n* = 8) and generally enjoyed eating (100%, *n* = 13). Piercy et al. (2021) described older people with MCADD as having a strong dependence on food and snacks [40]. However, this could not be confirmed in this study. Dietary restriction in daily life was denied by 75% o the participants. This may be due to differences in the age groups (8–20 vs. 10–15 years) and regional differences between the study locations (Germany vs. United Kingdom), between the cohorts, associated metabolic adaptations, psychosocial changes, dietary experience and care.

The strict fasting periods and parental control of food intake in MCADD patients in the early years of life may potentially affect the development of self-regulated perception of hunger and satiety [17–19]. Piercy et al. (2017) observed that parents of children with MCADD promote risk-averse eating behaviours in their children due to fear of the consequences of inadequate energy intake [49]. This was confirmed by the parents interviewed in the present study, who paid strict attention to their children’s food intake. In a later study, Piercy et al. (2021) identified a pervasive fear of the potential consequences of not eating among their study participants [40]. However, the majority of participants interviewed for this study could not confirm that they experienced an excessive fear of hunger, although they did report trusting their body’s own signals. Only 23.1% shared the fars described by Piercy et al. (2021). The reasons for the discrepancy between Piercy et al.’s (2021) results and those of the collective studied are unclear. One possible reason could be the intensive nutritional care and support received, although this has not yet been sufficiently investigated. However, fear of the consequences of inadequate energy intake could lead to increased food intake [40]. In this context, the accompanying fear of being overweight and associated dieting behaviour, which is more prevalent among adolescents, plays an important role and is considered a risk factor for developing eating disorders [22, 50, 51]. In this study, 66.7% of the female participants hd previously attempted to lose weight, compared to 28.3% of the male participants. Aditionally, 66.7% of the female participants rported being afraid of gaining weight, compared to 14.3% of male participants. These ender differences in the present sample are consistent with population-representative data from the second wave of the Eating study as a KiGGS Module (EsKiMo II) [52]. Even if there are no differences between the results of the EsKiMo II and the results of the present study in terms of dietary experience, dietary restriction may have significant health consequences, especially with regard to fasting intolerance in people with MCADD.

### Impact on family life

Most parents (76.9%, *n* = 10) in this study feel that family life is affected, especially holiday planning. Other influencing factors mentioned by the interviewed parents are worries stemming from the disease (50%, *n* = 5), illness events (40%, *n* = 4) and daily life characterised by diet and fasting intolerance (40%, *n* = 4). In this context, the burden of dietary measures on family life was significantly higher in infancy (0–4 years) than in the at the time of data collection in this study. Piercy et al. (2017) confirmed that the burden on parents decreases as their children get older. This is associated with a reduction in stress, as well as in parents’ fears and worries, as they become more accustomed to the situation and more confident in their coping abilities. However, parents remain concerned about the potential risk of a metabolic crisis [49]. Controversly, Gramer et al. (2014) found that 75% o parents of children with metabolic diseases felt that family life was affected, even in cases with a favourable prognosis [53]. This statement can only be confirmed for the current MCADD cohort in infancy. The differences between infancy and the current situation can be explained by the fact that fasting periods can be extended with increasing age, i.e. dietary measures become more relaxed as parents adapt to the situation, gain confidence in their abilities and generally return to a more typical daily routine. This is consistent with the findings of Piercy et al. (2017), who demonstrated a significant improvement in everyday life with increasing age of the children with MCADD. In summary, both the restrictions in everyday life and the burden on family life during infancy were mainly due to the need to maintain a safe fasting interval. The incidence of illness was also a significant factor in family burden. Piercy et al. (2017) also identified these two factors as the main challenges in coping with MCADD, and as the basis for the perceived burden on families [49].

### Limitations

One of the limitations of this study is the small sample size. Recruitment was limited to one University Hospital. However, the Metabolic Centre in Muenster is one of the largest centres in Germany. In addition, the limited population due to the presence of a rare disease has to be taken into account. Another limitation is found in the questions regarding eating problems. These were selected to avoid asking for redundant information, reviewed through experts, two pre-tests (content validation) and based on validated criteria. Nevertheless, the specific combination used was not further independently validated, meaning the results can only show tendencies of abnormal eating behaviour [26–29]. It cannot be ruled out that the questions in the online questionnaire were interpreted differently, which could introduce bias. Comprehension problems were largely avoided in the telephone interviews, so the combination of both methods is a strength of this study. Other potential sources of response bias could include social desirability, retrospective questions and parental influence. In addition, the order of the questions was not randomised, so the ‘halo effect’ cannot be excluded entirely [54]. Additionally, open-ended free-text questions are considered a limitation because responses may vary in scope, depth, and clarity. This heterogeneity can complicate systematic analysis and lead to subjectivity during categorisation. However, they can also provide valuable qualitative depth and perspectives [55]. Furthermore, an objective clinical assessment was not included in the study since the data came from regular outpatient appointments. This restricts the possibility of standardising the data. However, without this data, it would not have been possible to provide a retrospective overview of weight development. In terms of statistical analysis, the small number of cases increases the risk of type II errors and false negative results [56]. This problem could be mitigated in future through large-scale multicentre studies and evaluations.

## Conclusion

In conclusion, this observational and exploratory sample (*n* = 13) showed no tendencies of abnormal eating behaviour associated with dietary management in MCADD. However, no gender differences were found for fear of weight gain and previous dieting attempts. Analysis of weight histories revealed an increased tendency towards obesity in individuals with MCADD. However, only a small proportion of participants disregarded their feelings of hunger and satiety, and were constantly preoccupied with food. Dietary management and the disease appear to have a considerable impact on upbringing and family life. Particularly in infancy, dietary measures and episodes of illness are perceived as stressful and restrictive for the family. As the child grows older and dietary restrictions are eased, both the individual and their family seem to experience substantial relief.

## Supplementary Information

Supplementary Material 1: S1 Questionnaires and scoring, S1.1 Online questionnaire, S1.2: Telephone interview; S2 Evaluation of the questions on eating behavior; S3 Weight progression depending on the age of the participants, Figure S3.1 Weight progression depending on the age of the male participants, Figure S3.2 Weight progression depending on the age of the female participants

## Data Availability

The raw data (German language) supporting the conclusions of this article will be made available upon request by the corresponding author (T.F.; tobias.fischer@fh-muenster.de).
