# Supplementary material for: Effects of dietary management for medium-chain acyl-CoA dehydrogenase deficiency (MCADD) on eating behaviour in childhood, adolescence and young adulthood
Source: BMC Nutr. 2025 Nov 14;11:213. doi: 10.1186/s40795-025-01209-9 (PMC12619415; doi:10.1186/s40795-025-01209-9)
Supplement: Supplementary file 1 — Supplementary Material 1: S1 Questionnaires and scoring, S1.1 Online questionnaire, S1.2: Telephone interview; S2 Evaluation of the questions on eating behavior; S3 Weight progression depending on the age of the participants, Figure S3.1 Weight progression depending on the age of the male participants, Figure S3.2 Weight progression depending on the age of the female participants [file 40795_2025_1209_MOESM1_ESM.docx]

**Effects of dietary interventions for medium-chain acyl-CoA dehydrogenase deficiency (MCADD) on eating behaviour in childhood, adolescence and young adulthood**

# **Supplementary Materials**

# **S1 Questionnaires and scoring**

## **S1.1 Online questionnaire**

Personal details

1. Date of birth (example: 20.09.1998): [number]

2. Gender: 🞎 female 🞎 male 🞎 diverse

3. Height [in centimeters; example: 160 cm]: [number]

4. Body weight [in kilograms; example: 65.5 kg]: [number]

5. Type of school:

🞎 primary school

🞎 secondary school

🞎 comprehensive school

🞎 grammar school

🞎 other

6. Parents' highest school-leaving qualification/education?

🞎 No school-leaving qualification

🞎 elementary school

🞎 secondary school

🞎 comprehensive/grammar school

🞎 university degree

🞎 apprenticeship

🞎 other

7. Number of siblings? [number]

8. Is there anyone else in the family who also suffers from MCAD deficiency (MCADD)?

🞎 yes 🞎 no

1. How many family members are affected? [number]
2. Who is affected (sister/brother etc.)? [free text]

9. Has MCADD been detected by newborn screening?

🞎 yes 🞎 no

1. If not, how old was the child when diagnosed? [number]
2. How did the diagnosis come about? [free text]

10. Is carnitine (Biocarn) taken?

🞎 yes 🞎 no

11. Were there any hospitalizations due to the MCADD?

🞎 yes 🞎 no

If yes, how many (estimated number)? [number]

12. Are there any other known chronic illnesses?

🞎 yes 🞎 no

If yes, which ones? [free text]

13. Do you currently have any health complaints?

🞎 yes 🞎 no

1. If yes, which ones? [free text]
2. How severe are the restrictions in everyday life due to the health complaints?

🞎 very severe 🞎 severe 🞎 moderate 🞎 slight 🞎 not at all

14. Do you do any sport?

🞎 yes 🞎 no

1. What kind of sport do you do? [free text]
2. How often [per week]? [number]
3. and for how long [per week]? [number]

Questions about eating behavior

|  | **Applies** | **Tends to apply** | **Tends not to apply** | **Does not apply** | **Do not know** |
| --- | --- | --- | --- | --- | --- |
| 15. I eat a wide variety of foods. |  |  |  |  |  |
| 16. I like to try new LM that I have never eaten before. |  |  |  |  |  |
| 17. I feel that food determines my life. |  |  |  |  |  |
| 18. I have the feeling that food rules my life. The topic of food annoys me. It's far too often about what I eat, when I eat and how much I eat. |  |  |  |  |  |
| 19. I like to eat. |  |  |  |  |  |
| 20. I eat even though I'm not hungry because I'm afraid that my body won't have enough energy/if I don't feel well/if I have to go to the doctor/hospital again. |  |  |  |  |  |
| 21. If I eat my meals too late, I feel shaky. |  |  |  |  |  |
| 22. If I'm hungry, I have to eat something straight away because otherwise I'm afraid I'll feel sick. |  |  |  |  |  |
| 23. I keep eating, even though I'm already full, because I think I have to eat it all. |  |  |  |  |  |
| 24. I trust my body to tell me when to eat. |  |  |  |  |  |
| 25. I trust my body to tell me when to stop eating. |  |  |  |  |  |
| 26. I have tried to lose weight before and therefore ate less. |  |  |  |  |  |
| 27. I am afraid of gaining weight. |  |  |  |  |  |
| 28. I allow myself to eat what I feel like eating in the moment. |  |  |  |  |  |

## **S1.2: Telephone interview**

**Questions about eating behavior**

29. Number of meals per day.

30. Number of snacks per day.

31. Who is with you at mealtimes and where do you eat? (Do you eat with the whole family or individually?)

32. Who is mainly responsible for preparing meals in your household?

33. Do you currently pay attention to anything in particular when eating?

🞎 yes 🞎 no

1. If yes, what do you pay attention to?

34. Does the illness restrict you in terms of eating behavior in everyday life?

🞎 severely (3) 🞎 moderately (2) 🞎 slightly (1) 🞎 not at all

1. and why?

35. To what extent does the special eating behavior put a strain on family life?

🞎 strongly 🞎 moderately 🞎 slightly 🞎 not at all

1. and why?

36. Do you try to avoid foods with a lot of fat?

🞎 yes 🞎 no

1. If yes, why?

37. Are there any forbidden foods that you do not allow yourself to eat?

🞎 yes (1) 🞎 no

1. If yes, which ones?
2. and why?

***Questions about the disease***

38. Can you explain what kind of disease MCADD is?

🞎 yes 🞎 rather yes 🞎 rather no 🞎 no

39. Do you know what you need to pay attention to in your diet due to MCADD?

🞎 yes 🞎 rather yes 🞎 rather no 🞎 no

***Questions about care***

40. How satisfied are you with the current care at the UKM?

🞎 very satisfied 🞎 fairly satisfied 🞎 somewhat satisfied 🞎 not at all satisfied

41. What would you wish for regarding the current care?

*Questions for parents*

***Questions about eating behavior***

42. Has the diagnosis of MCADD (had) an influence on the upbringing of your child?

🞎 yes 🞎 no

1. If yes, to what extent?

***Questions about the disease***

38. Can you explain what kind of disease MCADD is?

🞎 yes 🞎 rather yes 🞎 rather no 🞎 no

39. Do you know what you need to pay attention to in your diet due to MCADD?

🞎 yes 🞎 rather yes 🞎 rather no 🞎 no

43. Has the disease (had) an impact on family life?

🞎 yes 🞎 no

1. If yes, in what way?

44. Do you currently pay attention to anything in particular when eating?

🞎 yes 🞎 no

1. If yes, what do you pay attention to?
2. What was it like in infancy (0-4 years)? 🞎 yes 🞎 no
3. If yes, what was paid attention to?

45. Does your child's illness restrict you in terms of eating behavior in everyday life?

🞎 severely 🞎 moderately 🞎 slightly 🞎 not at all

1. and why?
2. How was it in childhood (0-4 years)?

🞎 strongly 🞎 moderately 🞎 slightly 🞎 not at all

1. And why?

46. To what extent does the special eating behavior put a strain on family life?

🞎 strongly 🞎 moderately 🞎 slightly 🞎 not at all

1. And why?
2. How was it in infancy (0-4 years)?

🞎 strongly 🞎 moderately 🞎 slightly 🞎 not at all

1. And why?

47. do you try to avoid foods with a lot of fat in your child's diet?

🞎 yes 🞎 no

1. If yes, why?

48. Are there any forbidden foods that you do not allow your child to eat?

🞎 yes 🞎 no

a) If yes, which ones and why?

**Questions about the disease**

49. How would you rate your level of knowledge about MCADD?

🞎 very good 🞎 good 🞎 moderate 🞎 poor

50. How would you rate your level of knowledge about dietary treatment?

🞎 very good 🞎 good 🞎 moderate 🞎 poor

Questions about care

51. How well did you initially feel cared for by UKM?

🞎 very good 🞎 good 🞎 not so good 🞎 bad 🞎 we were looked after elsewhere

52. What wishes do you have regarding the initial consultation?

53. How satisfied are you with the current care at the UKM?

🞎 very satisfied 🞎 fairly satisfied 🞎 somewhat satisfied 🞎 not satisfied at all

54. What would you wish for regarding the current care?

55. Do you have any experience with hospital stays outside the UKM?

🞎 yes 🞎 no

1. What experiences have you had?
2. How were the experiences?

🞎 rather positive 🞎 partly positive 🞎 partly negative 🞎 rather negative

*🛈 Scoring was defined as follows: 1 point = (1); 2 points = (2); 3 points = (3)*

# **S2 Evaluation of the questions on eating behavior**

***Comparison of responses to questions 15-28, 34 and 37 by age group and gender (n = 13).*** *Calculation of independence using the Fisher exact test.*

|  | **total** [%] (n = 13) | **age group** [%] | | **p-value** | **gender** [%] | | **p-value** |
| --- | --- | --- | --- | --- | --- | --- | --- |
|  |  | **8-13 yrs.**  (n = 5) | **14-20 yrs.**  (n = 8) |  | **female**  (n = 6) | **male**  (n = 7) |  |
| **Question 15: I eat a wide variety of foods.** | | | | | | | |
| (Rather) true | 84,6 | 60,0 | 100,0 | 0,333 | 100,0 | 71,4 | 1,000 |
| (Rather) not true | 7,7 | 20,0 | 0,0 |  | 0,0 | 14,3 |  |
| Do not know | 7,7 | 20,0 | 0,0 |  | 0,0 | 14,3 |  |
| **Question 16: I like to try new foods that I have never eaten before.** | | | | | | | |
| (Rather) true | 61,5 | 40,0 | 75,0 | 0,293 | 66,7 | 57,1 | 1,000 |
| (Rather) not true | 38,5 | 60,0 | 25,0 |  | 33,3 | 42,9 |  |
| **Question 17: I have the feeling that food controls my life.** | | | | | | | |
| (Rather) true | 38,5 | 40,0 | 37,5 | 1,000 | 50,0 | 28,6 | 1,000 |
| (Rather) not true | 53,8 | 40,0 | 62,5 |  | 50,0 | 85,7 |  |
| Do not know | 7,7 | 20,0 | 0,0 |  | 0,0 | 14,3 |  |
| **Question 18: The topic of food annoys me. It's far too often about what I eat, when I eat and how much I eat.** | | | | | | | |
| (Rather) true | 30,8 | 20,0 | 37,5 | 1,000 | 33,3 | 28,6 | 1,000 |
| (Rather) not true | 61,5 | 60,0 | 62,5 |  | 66,7 | 57,1 |  |
| Do not know | 7,7 | 20,0 | 0,0 |  | 0,0 | 14,3 |  |
| **Question 19: I like to eat.** | | | | | | | |
| (Rather) true | 100,0 | 100,0 | 100,0 | -* | 100,0 | 100,0 | -* |
| (Rather) not true | 0,0 | 0,0 | 0,0 |  | 0,0 | 0,0 |  |
| **Question 20: I eat even though I'm not hungry because I'm afraid that my body won't have enough energy/otherwise I'll feel bad/I'll have to go to the doctor/hospital again.** | | | | | | | |
| (Rather) true | 23,1 | 0,0 | 37,5 | 0,491 | 33,3 | 14,3 | 1,000 |
| (Rather) not true | 69,2 | 80,0 | 62,5 |  | 66,7 | 71,4 |  |
| Do not know | 7,7 | 20, | 0,0 |  | 0,0 | 0,0 |  |
| **Question 21: If I eat my meals too late, I feel shaky.** | | | | | | | |
| (Rather) true | 23,1 | 20,0 | 25,0 | 1,000 | 16,7 | 28,6 | 1,000 |
| (Rather) not true | 76,9 | 80,0 | 75,0 |  | 83,3 | 71,4 |  |
| **Question 22: When I'm hungry, I have to eat something straight away because otherwise I'm afraid I'll feel sick.** | | | | | | | |
| (Rather) true | 30,8 | 40,0 | 25,0 | 1,000 | 33,3 | 28,6 | 1,000 |
| (Rather) not true | 69,2 | 60,0 | 75,0 |  | 66,7 | 71,4 |  |
| **Question 23: I keep eating even though I'm already full because I think I have to eat all this food to feel good.** | | | | | | | |
| (Rather) true | 15,4 | 20,0 | 12,5 | 1,000 | 16,7 | 14,3 | 1,000 |
| (Rather) not true | 84,6 | 80,0 | 87,5 |  | 83,3 | 85,7 |  |
| **Question 24: I trust my body to tell me when to eat.** | | | | | | | |
| (Rather) true | 61,5 | 60,0 | 62,5 | 1,000 | 50,0 | 71,4 | 0,545 |
| (Rather) not true | 23,1 | 20,0 | 25,0 |  | 33,3 | 14,3 |  |
| Do not know | 15,4 | 20,0 | 12,5 |  | 16,7 | 14,3 |  |
| **Question 25: I trust my body to tell me when to stop eating.** | | | | | | | |
| (Rather) true | 69,2 | 80,0 | 62,5 | 1,000 | 50,0 | 85,7 | 0,523 |
| (Rather) not true | 23,1 | 20,0 | 25,0 |  | 33,3 | 14,3 |  |
| Do not know | 7,7 | 0,0 | 12,5 |  | 16,7 | 0,0 |  |
| **Question 26: I have tried to lose weight before and have therefore eaten less.** | | | | | | | |
| (Rather) true | 46,2 | 40,0 | 50,0 | 1,000 | 66,7 | 28,6 | 0,286 |
| (Rather) not true | 53,8 | 60,0 | 50,0 |  | 33,3 | 71,4 |  |
| **Question 27: I am afraid of putting on weight.** | | | | | | | |
| (Rather) true | 38,5 | 40,0 | 37,5 | 1,000 | 66,7 | 14,3 | 0,242 |
| (Rather) not true | 53,8 | 40,0 | 62,5 |  | 33,3 | 71,4 |  |
| Do not know | 7,7 | 20,0 | 0,0 |  | 0,0 | 14,3 |  |
| **Question 28: I allow myself to eat what I feel like eating in the moment.** | | | | | | | |
| (Rather) true | 92,3 | 80,0 | 100,0 | -* | 100,0 | 85,7 | -* |
| (Rather) not true | 0,0 | 0,0 | 0,0 |  | 0,0 | 0,0 |  |
| Do not know | 7,7 | 20,0 | 0,0 |  | 0,0 | 14,3 |  |
| **Question 34: Does the illness restrict you in terms of eating behavior in everyday life? And if so, what restricts you?** | | | | | | | |
| A little | 25,0** | 0,0** | 37,5 | 0,491 | 16,7 | 33,33** | 1,000 |
| Not at all | 75,0** | 100,0** | 62,5 |  | 83,3 | 66,7** |  |
| **Question 37: Are there any forbidden foods that you do not allow yourself to eat?** | | | | | | | |
| Yes | 100,0** | 100,0** | 100,0 | -* | 100,0 | 100,0** | -* |
| No | 0,0** | 0,0** | 0,0 |  | 0,0 | 0,0** |  |

*p < 0.05 = significant*

** no calculation possible as only one response category is represented in the responses*

*** due to missing response n -1*

# **S3 Weight progression depending on the age of the participants**

***Figure S3.1*** Weight progression depending on the age of the male participants (0-20 years, n = 7). Compared to the reference curves of the percentiles according to Kromeyer-Hauschild et al. 2001

***Figure S3.2*** Weight progression depending on the age of the female participants (0-19 years, n = 6). Compared to the reference curves of the percentiles according to Kromeyer-Hauschild et al. 2001
